# Comparative genomics reveals molecular mechanisms underlying health and reproduction in cryptorchid mammals

**DOI:** 10.1186/s12864-021-08084-1

**Published:** 2021-10-26

**Authors:** Simin Chai, Xin Huang, Tianzhen Wu, Shixia Xu, Wenhua Ren, Guang Yang

**Affiliations:** School of Life Sciences, Nanjing Normal University, Nanjing, 210023 Jiangsu China

**Keywords:** Testicular position, Gubernaculum, Cancer resistance, Positive selection, Rapid evolution

## Abstract

**Background:**

Mammals have wide variations in testicular position, with scrotal testes in some species and ascrotal testes in others. Although cryptorchidism is hazardous to human health, some mammalian taxa are natural cryptorchids. However, the evolution of testicular position and the molecular mechanisms underlying the maintenance of health, including reproductive health, in ascrotal mammals are not clear.

**Results:**

In the present study, comparative genomics and evolutionary analyses revealed that genes associated with the extracellular matrix and muscle, contributing to the development of the gubernaculum, were involved in the evolution of testicular position in mammals. Moreover, genes related to testicular position were significantly associated with spermatogenesis and sperm fertility. These genes showed rapid evolution and the signature of positive selection, with specific substitutions in ascrotal mammals. Genes associated with testicular position were significantly enriched in functions and pathways related to cancer, DNA repair, DNA replication, and autophagy.

**Conclusions:**

Our results revealed that alterations in gubernaculum development contributed to the evolution of testicular position in mammals and provided the first support for two hypotheses for variation in testicular position in mammals, the “cooling hypothesis”, which proposes that the scrotum provides a cool environment for acutely heat-sensitive sperm and the “training hypothesis”, which proposes that the scrotum develops the sperm by exposing them to an exterior environment. Further, we identified cancer resistance and DNA repair as potential protective mechanisms in natural cryptorchids. These findings provide general insights into cryptorchidism and have implications for health and infertility both in humans and domestic mammals.

**Supplementary Information:**

The online version contains supplementary material available at 10.1186/s12864-021-08084-1.

## Background

**Fig. 1:**
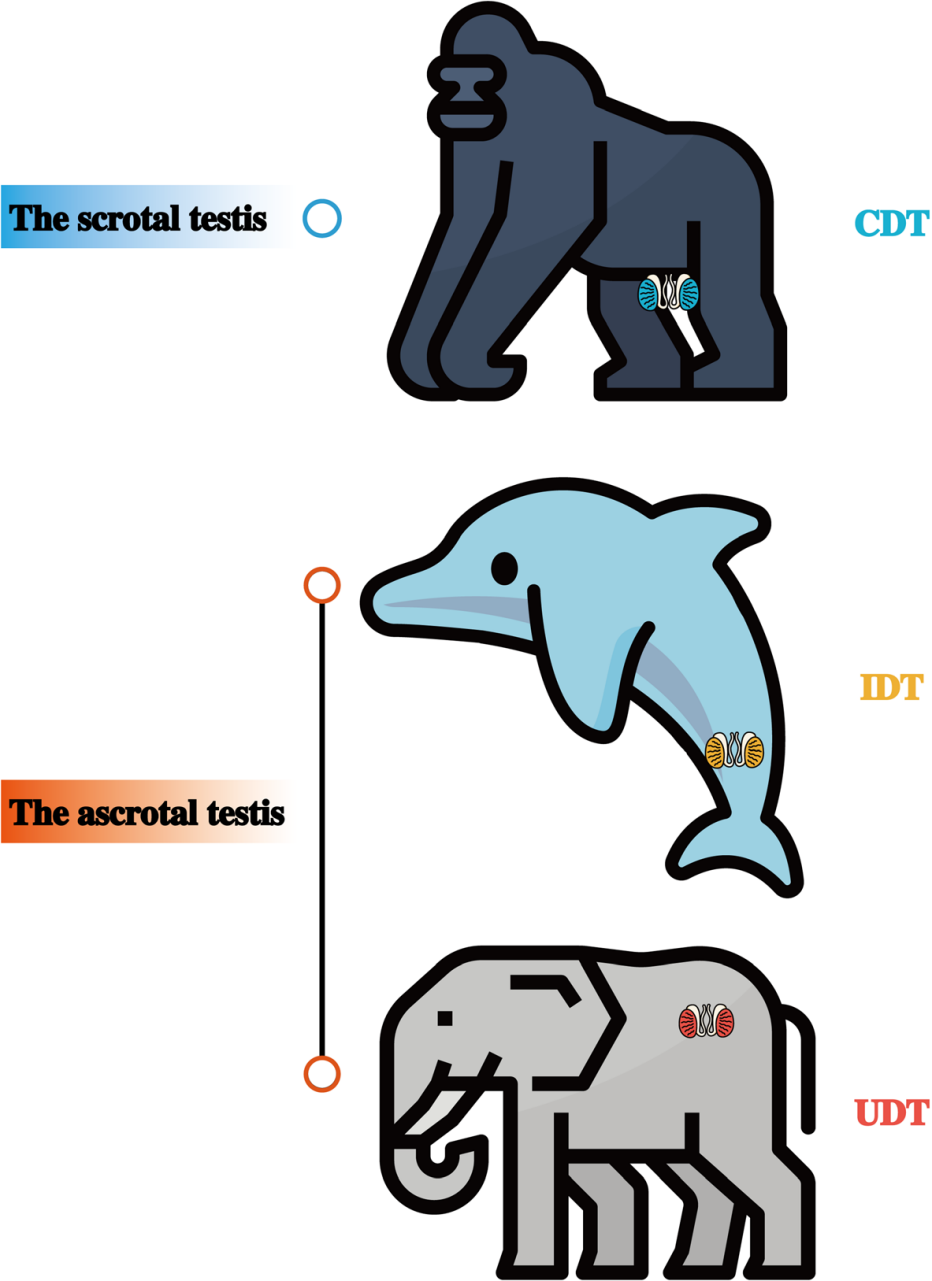
Variation in testicular position in mammals. CDT, completely descended testes; IDT, incompletely descended testes; UDT, undescended testes. Animal icons are from https://www.flaticon.com/

The diversity of testicular positions is one of the most remarkable traits in mammals [1]. Most mammals develop a pair of scrotal testes descending from the embryonic kidney region into scrotal sacs, which leave the body core, while a number of other mammals have ascrotal testes still located in the body core [2]. The scrotal testes exhibit complete descent (CDT), whereas ascrotal testes can be further classified into incompletely descended testes (IDT) in the inguinal region of the lower abdomen and the undescended testes (UDT) around the kidney (Fig. 1) [1, 3, 4]. For example, primates and most rodents carry scrotal CDT, whereas some highly specialized aquatic mammals, such as cetaceans, sirenians, and some bats, have ascrotal IDT, and monotremes and most afrotherians have UDT [2, 5].

From an adaptive evolution perspective, the diverse testicular positions in different groups of mammals benefits the adaptation to their unique environments and lifestyles. For example, the ascrotal testis is accompanied with streamlined body shape in many aquatic taxa (e.g., true seals, cetaceans and manatees) [1]. Specifically, a number of hypotheses have been proposed to explain the variation in testicular position among mammals, although none fully explains the observed variation. Based on the anatomical features that a pair of extracorporeal scrotums provide for thermoregulation (e.g., thin scrotal skin with sweat glands and fine hairs, tunica dartos, pampiniform plexus, cremaster muscle and absence of adipose tissue) [6–8], the ‘cooling hypothesis’ [9] states that scrotal CDT provide lower temperatures, which are beneficial for spermatogenesis. Short [10] extended this hypothesis and suggested that a cooler scrotal testis could be beneficial for maintaining the mutation rate in the male germ line at an acceptable level, without conferring an advantage for spermatogenesis. According to the ‘display hypothesis’ proposed by Portmann [11], the externally placed scrotal testis plays a role in sexual signaling and sex recognition. Additionally, the ‘training hypothesis’ [12] argues that the scrotum exposes the sperm to a physiologically hostile environment due to the finding that poor blood supply was provided for the scrotal testis. This physiologically hostile environment trained and screened few but high-quality sperms for the ultimate task – fertilization. From another perspective, the ‘galloping hypothesis’ [13, 14] proposes that the scrotal testis originated from galloping, jumping, leaping, and similar movements; the externalization of the testes protected the male gonads from fluctuations in intraabdominal pressures.

However, each hypothesis has been debated over decades. For instance, the cooling hypothesis has been argued against on the grounds that the scrotal testis is not obligatory for all mammals. Because, on the one hand, some (such as the hedgehog) have a low core body temperature; on the other hand, cetaceans and true seals are able to ease the thermoregulatory threat to a certain extent benefitting from the reproductive countercurrent heat exchangers (CCHEs) and venous system deep within the caudal abdominal cavity, respectively [15, 16]. With respect to the display hypothesis, the sexual signal function of the scrotal testis is limited to primates [5, 10, 11, 17, 18].

In general, testicular descent in scrotal CDT and ascrotal IDT mammals begins by the contraction of a cordlike ligament, the gubernaculum [5, 19]. During embryonic development, the gubernaculum develops from a mesenchymal core plus a muscular outer layer into a striated muscle bundle wrapped by extracellular matrix (ECM) [20–22]. Both in humans and domestic mammals, the failure of testicular descent results in cryptorchidism, a developmental defect related to various dysfunctions, such as asthenospermia, germ cell maldevelopment, and an elevated risk of testicular malignancy [7, 23–26]. Natural cryptorchid (ascrotal IDT and UDT) mammals do not show deficiencies in reproductive and general health. However, the genetic mechanisms by which these taxa avoid the health issues observed in humans with cryptorchidism are unclear.

The increasing availability of mammalian genome data provides an opportunity to explore the genetic factors involved in the evolution of diverse testicular positions and related health-supporting processes. In the present study, we used a comparative genomics approach to evaluate the evolution of gene families and one-to-one orthologs in 30 representative mammals, including natural cryptorchid species and scrotal mammals covering a broad taxonomic range. In particular, our findings and the detailed analysis of the molecular mechanisms underlying health in natural cryptorchid mammals provide a theoretical basis for the maintenance of male reproductive health in mammals.

## Results

### Expansion and contraction of gene families in ascrotal mammals

**Fig. 2:**
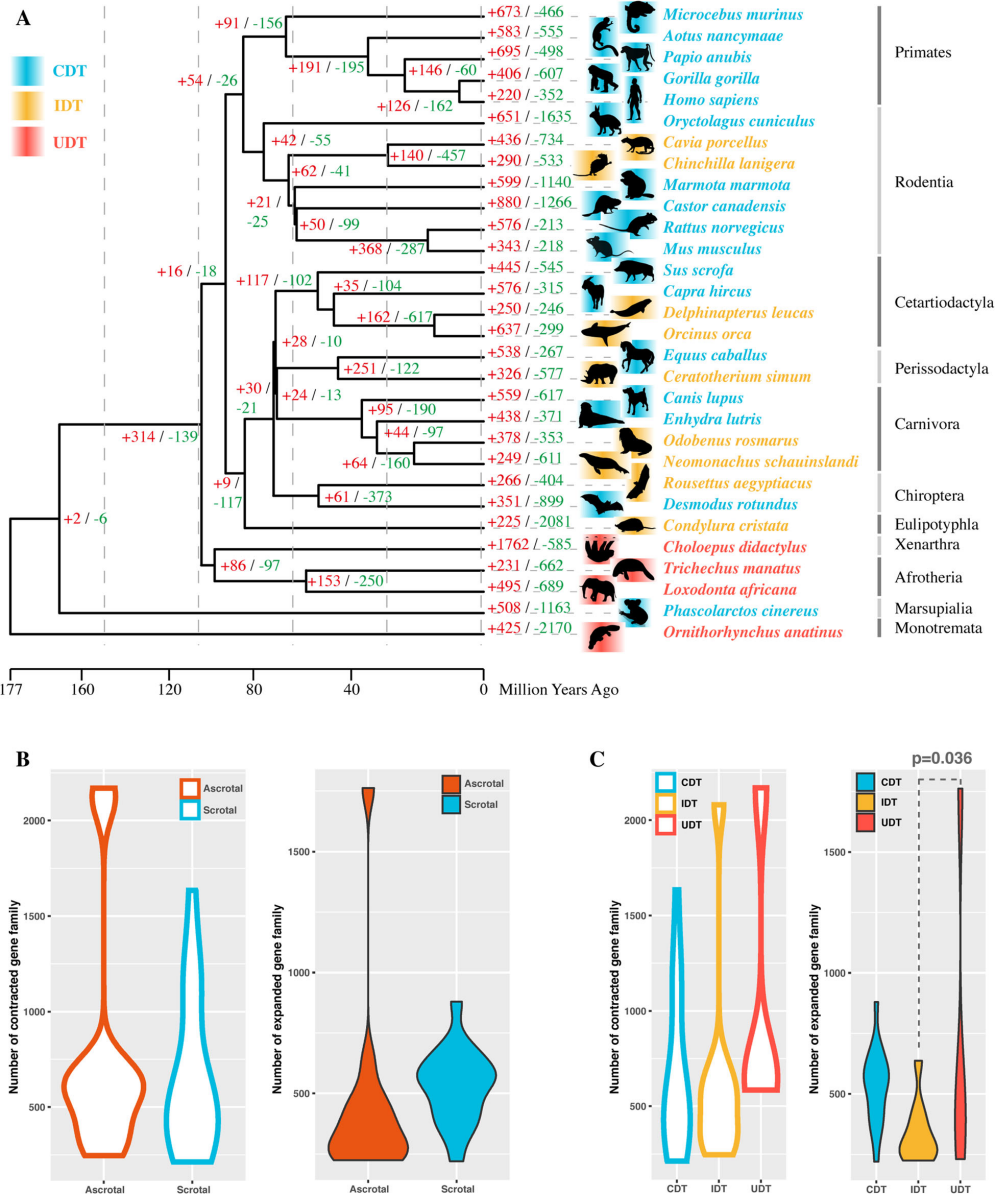
Gene family expansion and contraction. **A** Gene family expansion and contraction across a phylogenetic tree of 30 mammals. The numbers of expanded and contracted gene families in each species are shown in red and green, respectively. Scrotal CDT species are indicated by blue shading; ascrotal IDT and UDT species are indicated by yellow and red shading, respectively. Silhouettes of mammalian species are reproduced from http://phylopic.org/ under a Public Domain or Creative Commons license. **B** Phylogenetic ANOVA of the numbers of expanded and contracted gene families in ascrotal and scrotal mammals. **C** Phylogenetic ANOVA of the numbers of expanded and contracted gene families in CDT, IDT, and UDT mammals. Only the number of gene family expansions in IDT and UDT were significantly different (*p-value* = 0.036)

The evolution of gene family could be one of the contributing forces of specifically phenotypic adaptation [27]. We found that gene family size differed substantially among the 30 representative mammals examined in this study (Fig. 2A). And differences in gene family expansion and contraction were not related to differences in testicular position except for the difference between IDT and UDT mammals in gene family expansion (*p-value* = 0.036) (Fig. 2B, C). Three gene families, i.e., the Neurexophilin and PC-esterase domain (NXPE), olfactory receptors, and histone H2A family, shared similar patterns of expansion in at least 6 of 13 ascrotal species (Fig. S2).

### Association between gene evolution and mammalian testicular position

Gene-phenotype coevolution analysis by implementing the Phylogenetic Generalized Least Squares (PGLS) regression identifies molecular evolutionary correlates between genetic variants and different testicular positions [28]. Our PGLS analysis based on the 5333 one-to-one orthologs in 30 representative mammalian genomes identified 437 genes associated with the two-class testicular position (i.e., ascrotal and scrotal and 576 genes associated with the three-class testicular position, i.e., CDT, IDT, and UDT) (*p-value.all* < 0.05) (Table S4). After the two-step calibration procedure, 654 genes were confirmed to be associated with mammalian testicular positions (*p-value.max* < 0.05), including 424 and 410 genes associated with two-class and three-class testicular position, respectively. Among these, 180 genes were significantly associated with both two-class and three-class testicular position (Table S5).

**Fig. 3:**
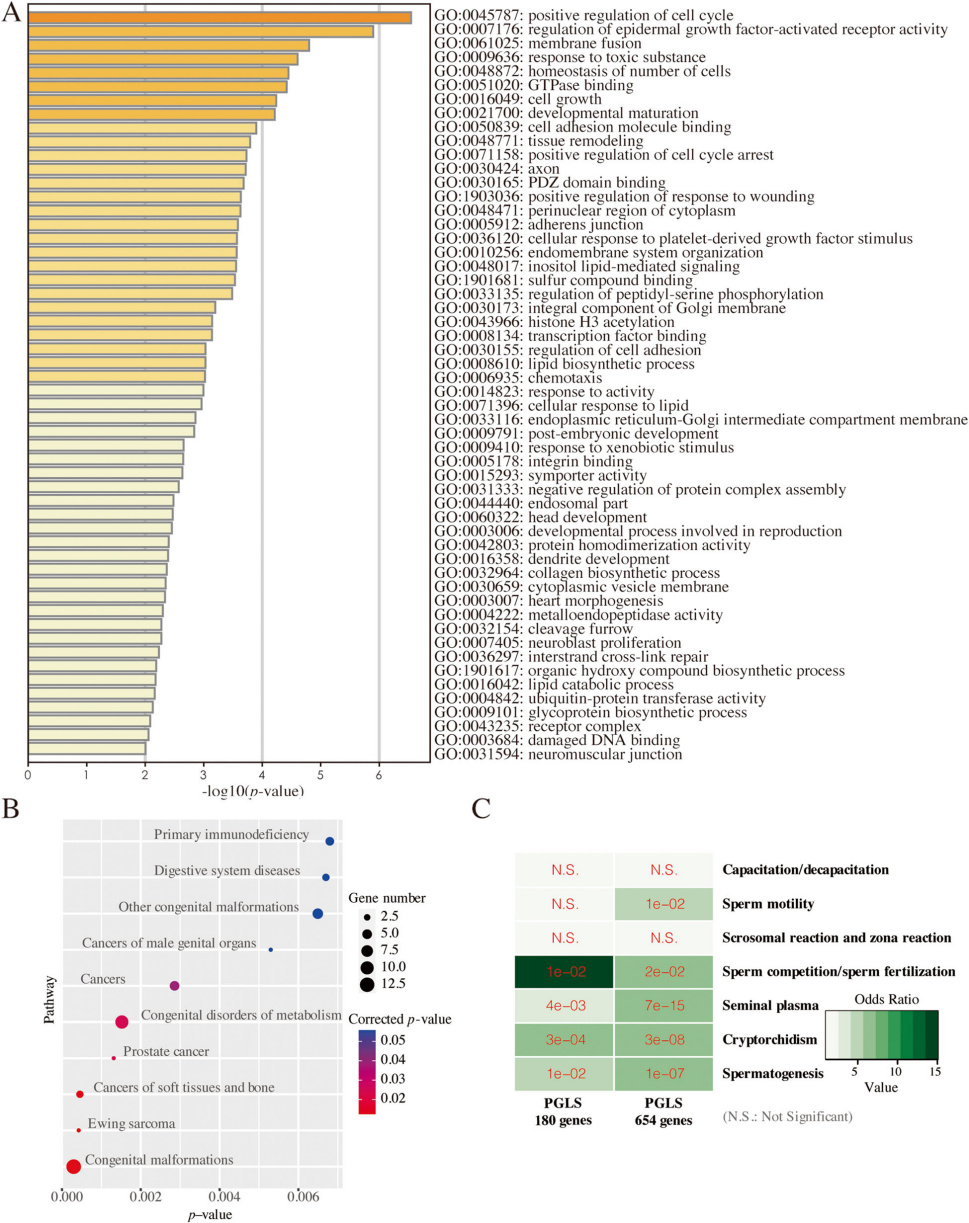
Function and pathway enrichment analyses and gene overlap analyses of testicular position-associated genes. **A** Top 100 GO enrichment terms for testicular position-associated genes using Metascape. **B** Top 10 enriched KEGG disease pathway with KOBAS. **C** Gene overlap test between the testicular position-associated genes and genes related to different sperm functions, spermatogenesis, and cryptorchidism

A Gene Ontology (GO) enrichment analysis performed using Metascape showed that these aforementioned 180 genes, which were significantly associated with both classifications of testicular position, were significantly enriched in terms related to GTPase binding, cell cycle, histone, DNA repair, reproduction, and spermatogenesis (Fig. 3A; Table S6). In the Kyoto Encyclopedia of Genes and Genomes (KEGG) disease enrichment analysis, the genes were significantly enriched in the terms ‘cancers of soft tissues and bone’ and ‘cancers of male genital organs’ (Fig. 3B; Table S7). Similarly, 654 testicular position-associated genes were also enriched in functions related to the cell cycle, GTPase binding, and apoptosis (Tables S8, and S9).

An overlap analysis indicated that 180 and 654 testicular position-associated genes are significantly involved in cryptorchidism (*p-value* = 3e-04 and 3e-08, respectively), spermatogenesis (*p-value* = 1e-02 and 1e-07, respectively), seminal plasma (*p-value* = 4e-03 and 7e-15, respectively), and sperm competition and fertilization (*p-value* = 1e-02 and 2e-02, respectively) (Fig. 3C). In addition, the 654 genes were also involved in sperm motility (*p-value* = 1e-02). These results suggested that the observed relationship between testicular position-associated genes and sperm function, spermatogenesis, and cryptorchidism is unlikely the result of chance.

### Specific amino acid substitutions in ascrotal mammals

**Fig. 4:**
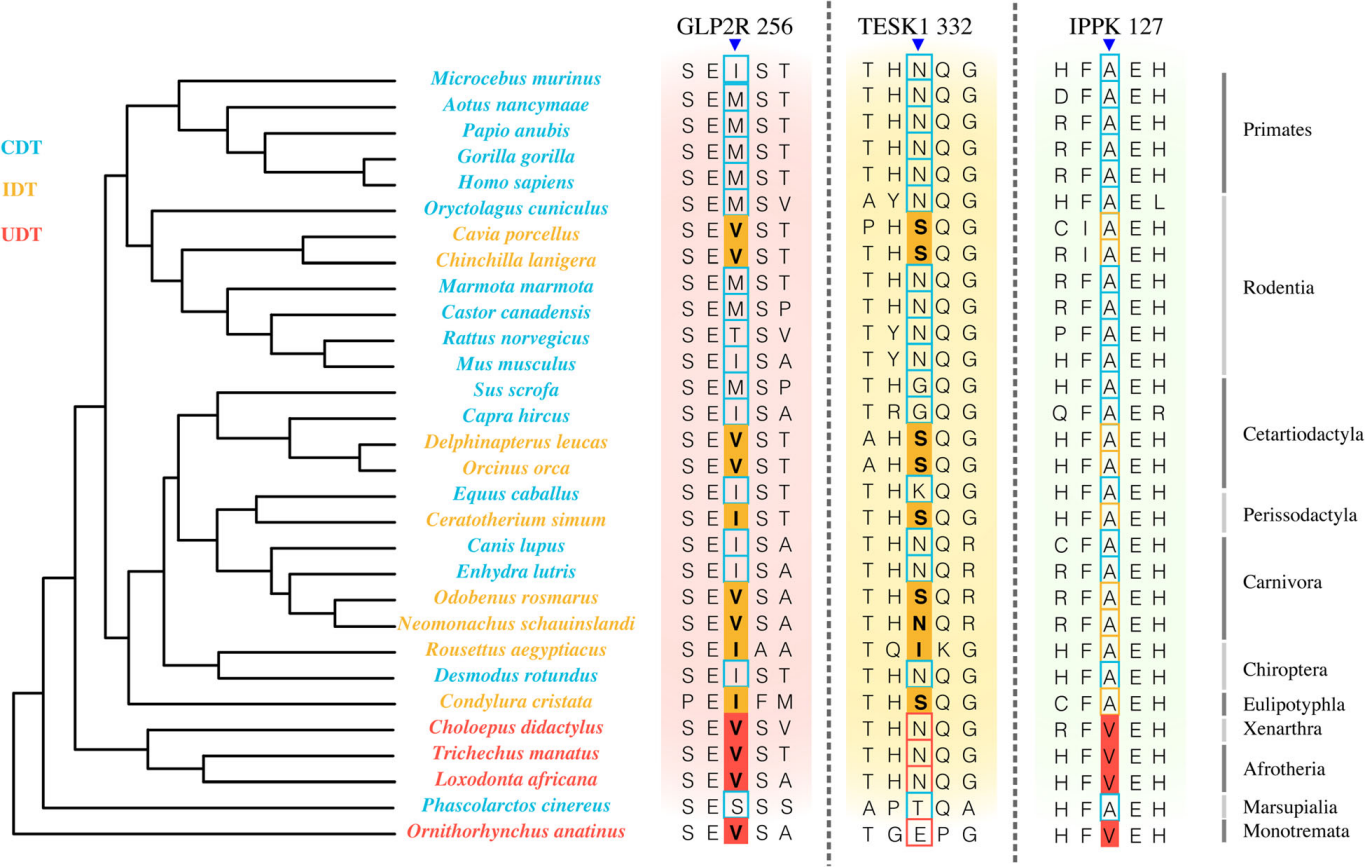
Specific amino acid substitutions in ascrotal mammals. From left to right, the three alignments illustrate the specific amino acid substitutions in ascrotal mammals (GLP2R M256V), in IDT mammals (TESK1 N332S/I), and in UDT mammals (IPPK A127V)

The fixed amino acid replacements in certain group of mammals might contribute to the explanation underlying a specific phenotype [29]. Among 5333 proteins and 3,585,819 amino acid positions, only one ascrotal mammal-specific amino acid substitution was identified in Glucagon Like Peptide 2 Receptor (GLP2R). GLP2R M256V was detected in most ascrotal species, including all four UDT and six of nine IDT species (Fig. 4). Moreover, Testis Associated Actin Remodeling Kinase 1 (TESK1) N332S/I was detected in eight out of nine IDT mammals, showing some degree of differentiation in this group (Fig. 4).

For UDT mammals, we identified 715 substitutions in 589 proteins (Fig. 4; Table S10). GO and KEGG enrichment analyses indicated that the genes encoding these 589 proteins were significantly overrepresented in pathways associated with DNA repair and functional terms related to DNA repair and replication as well as ECM and muscle development (Fig. S3, and S4).

### Rapid evolution and positive selection in genes in ascrotal mammals

**Fig. 5:**
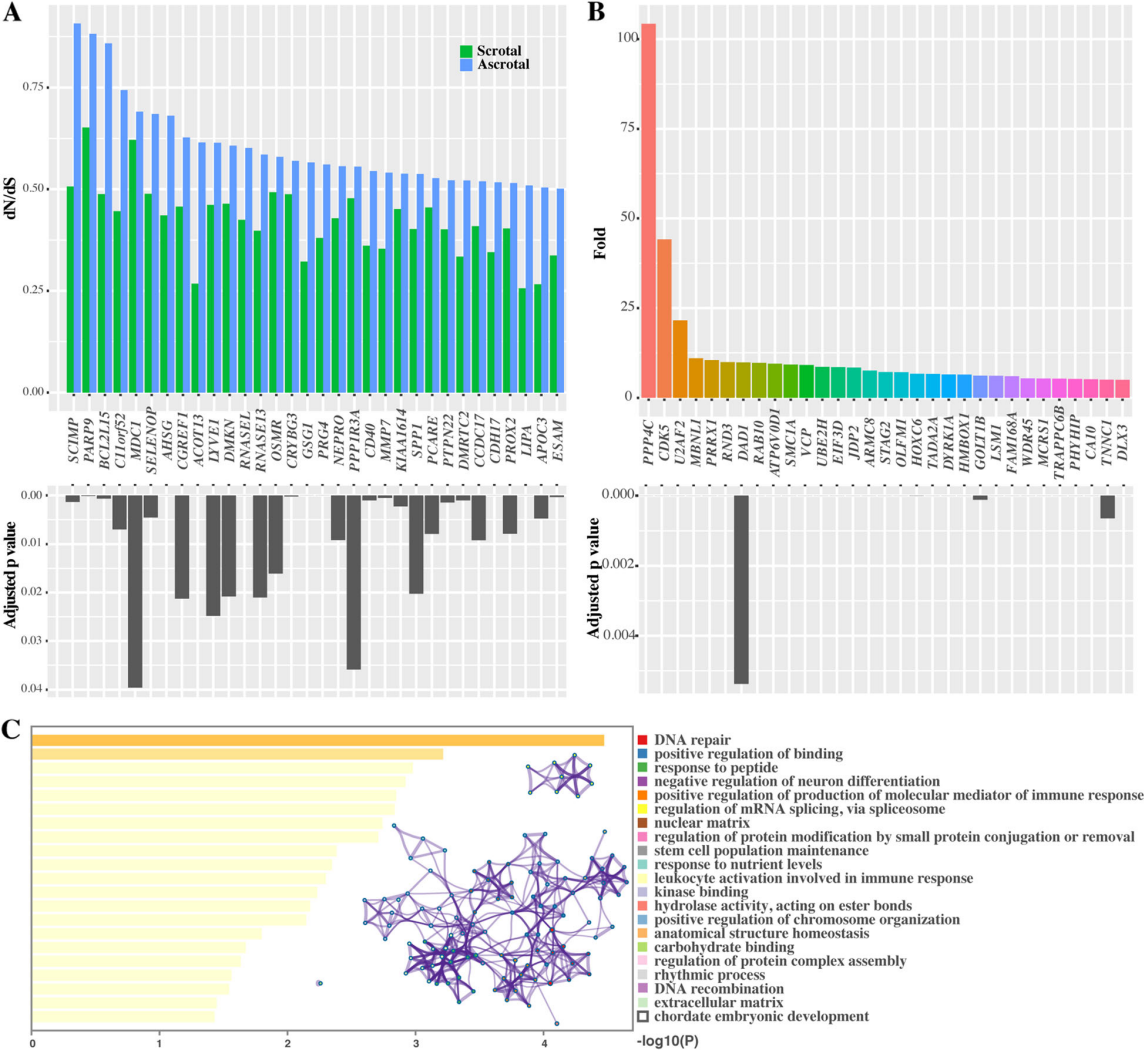
REGs in ascrotal mammals. **A** Thirty-one REGs in ascrotal mammals with ω_ascrotal_ > 0.5 and ω_ascrotal_ > ω_scrotal_ (adjusted *p-value* < 0.05). **B** Thirty-one REGs in ascrotal mammals with ω_ascrotal_/ω_scrotal_ > 5 (adjusted *p-value* < 0.05). **C** Heatmap and closely interactional clusters of top GO enrichment terms for 62 REGs in ascrotal mammals

The positive selection and rapid evolution of particular genes are essential for the phenotypic adaptation [30]. In the present study, after correction for multiple testing by false discovery rate (FDR) (adjusted *p-value* < 0.05), branch model analyses with codeml identified 62 rapidly evolving genes (REGs) in ascrotal mammals. Among them, 31 genes had ω_ascrotal_/ω_scrotal_ > 5 and the other 31 genes had ω_ascrotal_ > 0.5 and ω_ascrotal_ > ω_scrotal_ (Fig. 5A, B), suggesting differential selection pressures on mammals with different testicular positions. In addition, enrichment analyses indicated that REGs of ascrotal mammals were significantly overrepresented in DNA repair, DNA replication, and ECM (Fig. 5C).

The more stringent branch-site model identified 71 positively selected genes (PSGs) in ascrotal mammals (Table S11). These PSGs were significantly overrepresented in signaling pathways associated with cancer, chromatin modification, DNA repair, and autophagy (Table S12).

## Discussion

### Essential components of the gubernaculum contributed to the evolution of testicular position

For most scrotal mammals, a two-step testicular descent led to the development of a pair of scrotal testes, including (1) transabdominal descent and (2) inguino-scrotal descent [31]. A mechanical controller, the gubernaculum, connects with and pulls the testes. Consequently, a failure of gubernaculum development could lead to abnormal testicular descent in humans and mice [20].

Muscle tissues and abundant ECM are the primary components of the gubernaculum. During early development, the gubernaculum is composed of a mesenchymal core and a muscular outer layer [32]. The gubernaculum swells and is wrapped with massive ECM, probably due to increased expression of proteoglycans and glycosaminoglycan side chains [21].

We found that genes associated with testicular position, genes harboring UDT mammal-specific amino acid substitutions, and REGs of ascrotal mammals were significantly enriched in functional terms related to cell polarity, actin filament polymerization, muscle development, and ECM (Figs. 3A, and 5C, S3, S4; Table S6). These results suggested that genes related to ECM and muscle, which are essential for development of the gubernaculum, contribute to the evolutionary changes in testicular positions during mammalian evolution.

For example, we detected a UDT mammal-specific amino acid substitution (H979N) in Laminin Subunit Beta 1 (LAMB1) (Table S10), a member of the extracellular matrix glycoprotein family involved in cell adhesion, growth migration, and differentiation. H979N is located in an important Laminin EGF-like domain and participates in a disulfide bond maintaining the correct conformation of LAMB1 [33]. Further, functional prediction using HOPE [34] suggested that the mutant (asparagine) was smaller than the wild type (histidine), which might lead to loss of LAMB1 interactions and abolish its function (Fig. S5). C481F in another Laminin EGF-like domain is a deleterious mutation affecting protein function [35], and a mutation in LAMB1 (I1620T) has been reported in a male infant diagnosed with hydranencephaly along with cryptorchidism [36]. For these reasons, we hypothesized that H979N alters the ECM production capacity in UDT mammals. However, the functional effect of this substitution and its influence on testicular positions should be verified by further functional experiments. Similarly, Alpha 2-HS Glycoprotein (*AHSG*) was identified as a REG in ascrotal mammals (Fig. 5). This gene encodes an ECM component and a microdeletion including *AHSG* was associated with unilateral cryptorchidism [37], suggesting that it is involved in the process of testicular descent.

The swelling reaction and elongation of the gubernaculum are triggered by Insulin Like 3 (INSL3) [6]. Human cryptorchidism may be related to mutations in downstream signaling factors activated by INSL3 and its receptor [38]. Glucagon Like Peptide 2 Receptor (*GLP2R*) is in the same super pathway as *INSL3* (Fig. S6) and contained an ascrotal mammal-specific substitution (M256V) (Fig. 4). *GLP2R* is also an epididymis secretory sperm binding gene, and it is highly expressed in the testis but not in the female reproductive organs [39]. The M256V substitution was located in the extracellular region, 7tm_2 (PF00002 in the Pfam database), an important domain for binding to large peptidic ligands. The smaller size of the mutant was predicted to lead to a loss of these interactions (Fig. S7) and thus cause functional alterations, supporting its potential role in the development of the gubernaculum and evolution of testicular position.

### Rapid evolution of sperm fertilization- and spermatogenesis-related genes supports the cooling hypothesis and training hypothesis

Although it has been an intriguing question for a long period of time, no consensus on the evolutionary mechanism driving differences in testicular positions in mammals has been reached. While a number of hypotheses have been put forward [9–14], the cooling hypothesis has received the most support because it effectively explains the function of the scrotum based on thermoregulation during spermatogenesis [4, 40]. However, most evidence in support of this hypothesis is based on physiological and ecological observations and inferences, with very little molecular evidence from an evolutionary perspective.

We found that divergence in a substantial number of genes related to spermatogenesis and sperm fertilization corresponded with the evolution of testicular position in mammals (Fig. 3), providing some new molecular insights into the cooling hypothesis and the training hypothesis. In scrotal mammals, the scrotum contributes to thermoregulation by the maintenance of a lower temperature than the body core, which is essential for spermatogenesis [41]. Although overheating harms normal spermatogenesis and male fertility [24] in mammals, this is not the case for natural cryptorchid mammals, especially for most ascrotal taxa with perfect homoiothermy and higher body core temperatures than the optimal condition for spermatogenesis [40], implying that an adaptive mechanism for spermatogenesis evolved in ascrotal mammals. Sixteen testicular position-associated genes including Intraflagellar Transport Associated Protein (*IFTAP*), Cation Channel Sperm Associated 1 (*CATSPER1*), BTB Domain Containing 18 (*BTBD18*), AlkB Homolog 5 RNA Demethylase (*ALKBH5*), Ovo Like Transcriptional Repressor 1 (*OVOL1*), VPS33B Interacting Protein Apical-Basolateral Polarity Regulator Spe-39 Homolog (*VIPAS39*), SKI Like Proto-Oncogene (*SKIL*), Solute Carrier Family 9 Member C1 (*SLC9C1*), Eukaryotic Translation Initiation Factor 5A2 (*EIF5A2*), Adenylate Cyclase 10 (*ADCY10*), Beta-1,4-N-Acetyl-Galactosaminyltransferase 1 (*B4GALNT1*), Activin A Receptor Type 2A (*ACVR2A*), Elongation Factor For RNA Polymerase II 3 (*ELL3*), Golgi Reassembly Stacking Protein 2 (*GORASP2*), Pleckstrin Homology Domain Containing A1 (*PLEKHA1*), and Spermatogenesis Associated 5 (*SPATA5*) were significantly related to spermatogenesis in an overlap analysis (Fig. 3C). Additionally, GORASP2 L336I, CATSPER1 I544V, BTBD18 K209R, and SPATA5 H811R were identified as UDT-specific substitutions (Table S10). *SPATA5* encodes a member of the ATPases associated with the diverse activities (AAA) protein family and is involved in mitochondrial morphogenesis during differentiation in spermatogenesis, which might be a compensatory mechanism for thermodynamically unfavorable conditions and decreased oxygen supply due to the blood–testis barrier [42]. Previously reported mutations (R784Q and A844V) located in or near the AAA domain disrupt the function of SPATA5 [42]. Interestingly, the SPATA5 H811R mutation detected in the present study was not only near the AAA domain but also introduced a radical change in amino acid residual charge from neutral to positive. This substitution was predicted to lead to the repulsion of interactions between SPATA5 and other molecules (Fig. S8). Presumably, genes and codons in ascrotal UDT mammals evolved to address the potential heat stress in internal testes.

Moreover, we identified an IDT mammal-specific substitution in TESK1 (N332S/I). This protein has roles in spermatogenesis and is associated with spermatogenic failure. The N332S/I mutation site is close to the protein kinase domain, and both mutants (isoleucine and serine) were predicted to be smaller and more hydrophobic than the wild type, resulting in a loss of hydrogen bonds and/or disruption in correct folding (Fig. S9). The structure and function of TESK1 may be influenced by this substitution, contributing to spermatogenesis in natural cryptorchid testes.

In addition to the cooling hypothesis, our evolutionary analyses provide some support for the training hypothesis. Compared with the ascrotal testis, sperms in the scrotal testis are exposed to a hostile environment (poor in oxygen and blood supply) to train for fertilization, the final task of gametes [12]. Despite the advantages of scrotal testes in mammals, their development is a complex process requiring energy and material expenses. Ascrotal mammals evolved an alternative strategy to resolve the lack of a training area (i.e., testicular externalization). We identified a number of testicular position-associated genes with beneficial roles in fertilization, e.g., sperm competition/fertilization, seminal plasma, and sperm motility (Fig. 3). For instance, MLX Interacting Protein Like (*MLXIPL*) encodes a transcription factor in the Myc/Max/Mad superfamily, with a basic helix-loop-helix and a Leucine-zipper domain required for dimerization and DNA binding. MLXIPL expression is significantly decreased in mice whose spermatogenesis and fertility are disturbed [43], and mutations in this gene are associated with Williams syndrome, characterized by cryptorchidism in male patients [44]. In the present study, the testicular position-associated gene *MLXIPL* showed a unique substitution (Q716R) in the important Leucine-zipper domain in UDT mammals. The arginine substitution was predicted to change the function of MLXIPL based on the larger volume and positive charge introduced by this residue (Fig. S10), consequently contributing to fertilization in ascrotal mammals.

*CATSPER1* is a voltage-gated calcium channel essential for successful sperm fertilization and is exclusively expressed in the testis. Sperm function in humans and fertilization in mice are inhibited in vitro when *CATSPER1* is blocked [45]. The distinct evolutionary trajectories of *CATSPER1* between ascrotal and scrotal mammals suggest that this gene is involved in adaptation to sperm competition in natural cryptorchid testes retained in the body core.

### Natural cryptorchid mammals show improved cancer resistance and DNA repair

In mammals, including humans, cancer is one of the most important issues affecting health and lifespan [46, 47]. In young men, testicular cancer is the most common malignancy, and cryptorchidism (undescended or maldescended testicles) confers a 2- to 4-fold increase in risk [48].

However, little is known about infertility and/or cancers (especially testicular cancer) in natural cryptorchid mammals. Our results provide new insights into the molecular mechanisms underlying cancer or malignancy resistance in cryptorchid mammals. In particular, genes associated with testicular position and PSGs in ascrotal mammals were significantly enriched in pathways related to cancer, especially cancers of male genital organs (Fig. 3B, Tables S7, and S12). This observation strongly suggested that the increased cancer resistance evolved in natural cryptorchid mammals. For example, the positively selected gene Bone Morphogenetic Protein 4 (*BMP4*) encodes a protein involved in multiple human cancers, especially cancer in male external genitalia, and cryptorchidism [49]. In addition, BMP4 was highly differentially expressed between the gubernaculum of wild-type and orl (a cryptorchid strain) fetal rats, implying its importance in the development of the gubernaculum and testicular descent [50]. The positively selected site (G44M) lies in the conserved propeptide and antibody binding region of BMP4, and mutations in the propeptide would influence the release of mature BMP4 [51]. Given that the expression of *BMP4* affects the biological behavior of tumors [51], the change in BMP4 quantity in ascrotal mammals might increase cancer inhibition.

Spermatogenesis, the core function of the key male reproductive organ, is a complex process involving cell differentiation, cell proliferation, and meiosis, in which DNA synthesis is indispensable. Notably, the testis and germ cells are sensitive and vulnerable to heat stress. The decreased DNA replication activity in testes of cryptorchid rats is related to the deleterious effects of elevated temperatures [52]. An elevation in DNA damage in sperm from subfertile men is influenced by (at least in part) cryptorchidism [53] because pathological cryptorchidism generally leads to higher temperatures of the testes [24]. Moreover, aberrant seminiferous epithelial cycles and abnormal autophagy have been observed in mice with surgery-induced cryptorchidism [54]. Thus, to retain genomic stability and repair replication errors, natural cryptorchid mammals may have developed mechanisms for improved DNA repair, optimal autophagy, and accurate DNA replication.

In this study, we obtained several lines of evidence for improved cancer resistance and DNA repair. First, there were more histone H2A family members in ascrotal mammals than in their scrotal counterparts (Fig. S2). These genes, with posttranslational modifications, play a key role in the regulation of chromatin structure as well as DNA repair [55]. Second, genes associated with testicular position and REGs in ascrotal mammals as well as genes possessing UDT mammal-specific amino acid substitutions were significantly enriched for functions in DNA repair and replication (Fig. 3A, S3 and S4; Tables S6 and S12). Third, PSGs in ascrotal mammals were enriched in autophagy pathways (Table S12).

For example, ABL Proto-Oncogene 1 Non-Receptor Tyrosine Kinase (*ABL1*) is a protooncogene and primarily contributes to the DNA damage response and autophagy. The protein had a UDT-specific amino substitution (P997Q) in its F-actin-binding domain (FABD). The function of this tyrosine kinase depends on FABD, which negatively regulates ABL1 [56]. In a mouse model, mutants of the FABD of ABL1 lead to changes in oncogenicity [57]. The protein with the substitution of the polar glutamine in UDT mammals was predicted to be larger and less hydrophobic than the protein with nonpolar proline, and this substitution would alter the special backbone conformation provided by the rigid proline (Fig. S11) [58]. Consequently, P997Q is highly likely to alter the binding of ABL1 to F-actin and other substrates in ascrotal mammals, resulting in an improved sensitivity to DNA damage in the internal testis, thereby contributing to reproduction and health.

## Conclusions

To clarify the molecular mechanisms underlying the evolution of diverse testicular positions in mammals and strategies for the maintenance of reproductive health in natural cryptorchid mammals, we surveyed 30 genomes from taxa covering the range of variation in testicular position. Our genome-wide analyses suggested that genes associated with muscle and ECM (involved in the development of the gubernaculum, an essential structure for testicular descent) contributed to the evolution of mammalian testicular position. In addition, gene family expansion in ascrotal mammals combined with the signal of positive selection, rapid evolution, and specific substitutions in ascrotal mammals indicated that cancer resistance and DNA repair contribute to the maintenance of health in natural cryptorchid mammals. We detected significant associations between testicular positions and the divergence of genes related to spermatogenesis and fertility, providing the first support for the cooling hypothesis and the training hypothesis from a molecular evolutionary perspective. In summary, the results of this study provide novel insights regarding the evolutionary strategies by which ascrotal mammals avoid the negative consequences of natural cryptorchidism.

## Methods

### Phenotypic and genomic data collection

The testicular position of 30 mammals representing a broad taxonomic range with high sequencing and assembly quality were collected from previous studies (Table S1) [1, 3, 12, 59].

Thirty reference genomes were downloaded from https://www.ncbi.nlm.nih.gov/genome (Table S1). The longest transcript for each gene with alternative splicing variants was chosen for analyses. Further, gene families and orthologs were called by OrthoFinder using an all-against-all BLASTP algorithm [60]. Gene family expansion and contraction were analyzed using CAFE (Computational Analysis of gene Family Evolution) [61]. The phylogeny used in CAFE (and in other subsequent evolutionary analyses such as PAML) was dated using TimeTree (http://www.timetree.org/) (Fig. S1) [62]. A total of 5334 one-to-one single copy protein-coding orthologs in the 30 representative mammals were generated.

To test if there is a difference in the extent of gene family expansion and contraction among mammals with different testicular positions, phylogenetic ANOVA was used (phylANOVA function in phytools package in R) [63], with a *p*-value cutoff of 0.05.

Codon-based alignments were generated using MACSE with default parameters [64]. Further, poorly aligned regions and gaps were trimmed using Gblocks [65]. Finally, 5333 alignments were generated; *TTN* was too long to align successfully. The 5333 aligned nucleotide sequences and gene information were provided in *figshare Dataset* (10.6084/m9.figshare.16565982.v1) and Table S2.

### Rapidly evolving and positively selected genes

Evolutionary rates (ω) estimated by the ratio of the rates of nonsynonymous (*d*_N_) and synonymous (*d*_S_) substitutions per site were computed using codeml in PAML [66], considering the rate of transitions and transversions and effects of codon usage. The fit of each hypothesis within a pair of nested models was compared using a likelihood ratio test (LRT). The Benjamini–Hochberg method [67] was applied to correct for multiple testing, and a false discovery rate cutoff of 0.05 was used.

To identify REGs in ascrotal mammals, the two-ratio model in PAML was employed [66]. The null hypothesis (one-ratio model) assumed that genes in scrotal and ascrotal species evolved with the same ω, while the alternative hypothesis set two different ω values for the foreground (i.e., the ascrotal) and background (i.e., the scrotal) species. Genes with ω_ascrotal_/ω_scrotal_ > 5, or ω_ascrotal_ > 0.5 and ω_ascrotal_ > ω_scrotal_ were considered REGs in ascrotal mammals.

To identify PSGs with ω > 1, a nested branch-site model was used. The branch-site model detected both genes and codons under positive selection in foreground species (i.e., the combined ascrotal mammals). Genes with a significant LRT (adjusted *p-value* < 0.05) and selected codons with posterior possibility > 0.8 were identified as positively selected.

### Association between root-to-tip ω and the testicular position

The root-to-tip ω value of a gene refers to the average of the accumulated ω extending from the last common ancestor to the extant species and is an index of selection, including the evolutionary history of a gene in a certain species [68, 69]. The evolutionary rate of each one-to-one ortholog was calculated using codeml in PAML [66] with a free-ratio model. In particular, branches with *d*_N_ or *d*_S_ values of < 0.0002 were excluded from subsequent analyses because substitution rates equal to or approaching zero would lead to exceedingly high or low ω values.

To evaluate the association between gene evolution and mammalian testicular positions, the calculated root-to-tip ω values were assigned to the working phylogenetic topology for a PGLS regression analysis [70] using the Caper package in R [71]. PGLS regression was performed for testicular positions of two (scrotal and ascrotal) and three classes (CDT, IDT, and UDT) against root-to-tip ω values.

For each gene, the strength of the correlation was evaluated by using an extra two-step calibration procedure, which is equal to or better than multiple testing correction [72]. Based on ‘*p-value.all*’ from the original regression analyses, ‘*p-value.robust*’ (from the re-calculation of regressions after discarding the largest residual error species) and ‘*p-value.max*’ (the maximum or least significant *p*-value from the third calculation of the regression after excluding each species one at a time) were evaluated. This procedure ensured that the correlation between genotypic and phenotypic data was generalizable and did not depend on a single species. *P-value.max* < 0.05 was chosen as the cutoff.

### Identification and functional prediction of specific amino acid substitutions

Specific substitutions were identified using FasParser [73] for the ascrotal (IDT + UDT), UDT, and IDT groups. Human canonical sequences in UniProt (https://www.uniprot.org/) [33] were taken as references for the locations of amino acids. HOPE was used to predict structural and functional effects of an amino acid substitution [34].

### Gene function and signaling pathway annotation and enrichment

The cryptorchidism-related gene list was obtained from the *Cryptorchidism Gene Database* [74]. Capacitation/decapacitation, sperm motility, sperm competition/sperm fertilization, acrosomal reaction, zona reaction, and spermatogenesis-related gene lists were gathered from Gene Ontology (http://geneontology.org/) [75, 76]. The sperm function genes for seminal plasma was obtained from GeneCards (https://www.genecards.org/) [77] with a relevance score > 2 as a cutoff. The relevant sperm function genes are provided in Table S3.

To test whether genes associated with mammalian testicular positions are involved in sperm functions, spermatogenesis, and cryptorchidism, gene overlap was evaluated using the GeneOverlap package in R [78]. Fisher’s exact test was used to test if two gene lists were independent. The odds ratio measured the strength of the association between two gene lists, where odds ratio > 1 indicated a strong association and odds ratio < 1 implied no association.

GO functional [79], KEGG pathway [80], and Reactome pathway [81] enrichment analyses were performed using Metascape setting with *Homo sapiens* as the “Input as species” and “Analysis as species” (http://metascape.org) [82] and KOBAS [83], setting *p-value* < 0.05 as a cutoff.

## Supplementary Information

**Additional file 1: Table S1**. Thirty representative mammals in the present study and their testicular positions. **Table S2**. The information of 5333 genes. **Table S3**. Lists of cryptorchidism, spermatogenesis and different sperm function-related genes. **Table S4**. Genes evolved associated with different testicular positions (*p*-value.all < 0.05). **Table S5**. Genes evolved associated with different testicular positions (*p*-value.max < 0.05). **Table S6**. GO enrichment of 180 testicular position-associated genes with Metascape. **Table S7**. KEGG disease enrichment of 180 testicular position-associated genes with KOBAS. **Table S8**. GO enrichment of 654 testicular position-associated genes with metascape. **Table S9**. KEGG enrichment of 654 testicular position-associated genes with metascape. **Table S10**. UDT mammal-specific amino acid substitutions. **Table S11**. PSGs in ascrotal mammals. **Table S12**. KEGG and reactome pathway enrichment of 71 PSGs in ascrotal mammals.

**Additional file 2: Fig. S1**. The phylogeny of 30 mammals used in CAFE (and in other subsequent evolutionary analyses such as PAML) using TimeTree (http://www.timetree.org/). **Fig. S2**. The expanded gene family shared in ascrotal mammals. **Fig. S3**. Heatmap of Top100 GO enrichment terms of 589 genes containing UDT mammal-specific amino acid substitutions. **Fig. S4**. Heatmap of KEGG pathway enrichment terms of 589 genes containing UDT mammal-specific amino acid substitutions. **Fig. S5**. Functional prediction of LAMB1 H979N. **Fig. S6**. Interaction between GLP2R and INSL3 from String (https://version11.string-db.org/). **Fig. S7**. Functional prediction of GLP2R M256V. **Fig. S8**. Functional prediction of SPATA5 H811R. **Fig. S9**. Functional prediction of TESK1 N332S/I. **Fig. S10**. Functional prediction of MLXIPL Q716R. **Fig. S11**. Functional prediction of ABL1 P997Q.

## Data Availability

All of the 5333 aligned sequences are available in the *figshare* Dataset (10.6084/m9.figshare.16565982.v1). Other data generated or analysed during this study are included in this published article and its supplementary information files.

## Abbreviations

CDT: Complete Descended Testes
IDT: Incompletely Descended Testes
UDT: Undescended Testes
ECM: Extracellular Matrix
NXPE: The Neurexophilin and PC-esterase Domain
PGLS: Phylogenetic Generalized Least Squares
GO: Gene Ontology
FDR: False Discovery Rate
REG: Rapidly Evolving Gene
PSG: Positively Selected Gene
LRT: Likelihood Ratio Test
