# Supplementary material for: Comparative genomics reveals molecular mechanisms underlying health and reproduction in cryptorchid mammals
Source: BMC Genomics. 2021 Oct 26;22:763. doi: 10.1186/s12864-021-08084-1 (PMC8547080; doi:10.1186/s12864-021-08084-1)
Supplement: Supplementary file 2 — Additional file 2: Fig. S1. The phylogeny of 30 mammals used in CAFE (and in other subsequent evolutionary analyses such as PAML) using TimeTree (http://www.timetree.org/). Fig. S2. The expanded gene family shared in ascrotal mammals. Fig. S3. Heatmap of Top100 GO enrichment terms of 589 genes containing UDT mammal-specific amino acid substitutions. Fig. S4. Heatmap of KEGG pathway enrichment terms of 589 genes containing UDT mammal-specific amino acid substitutions. Fig. S5. Functional prediction of LAMB1 H979N. Fig. S6. Interaction between GLP2R and INSL3 from String (https://version11.string-db.org/). Fig. S7. Functional prediction of GLP2R M256V. Fig. S8. Functional prediction of SPATA5 H811R. Fig. S9. Functional prediction of TESK1 N332S/I. Fig. S10. Functional prediction of MLXIPL Q716R. Fig. S11. Functional prediction of ABL1 P997Q. [file 12864_2021_8084_MOESM2_ESM.pdf]

Supplementary Figures

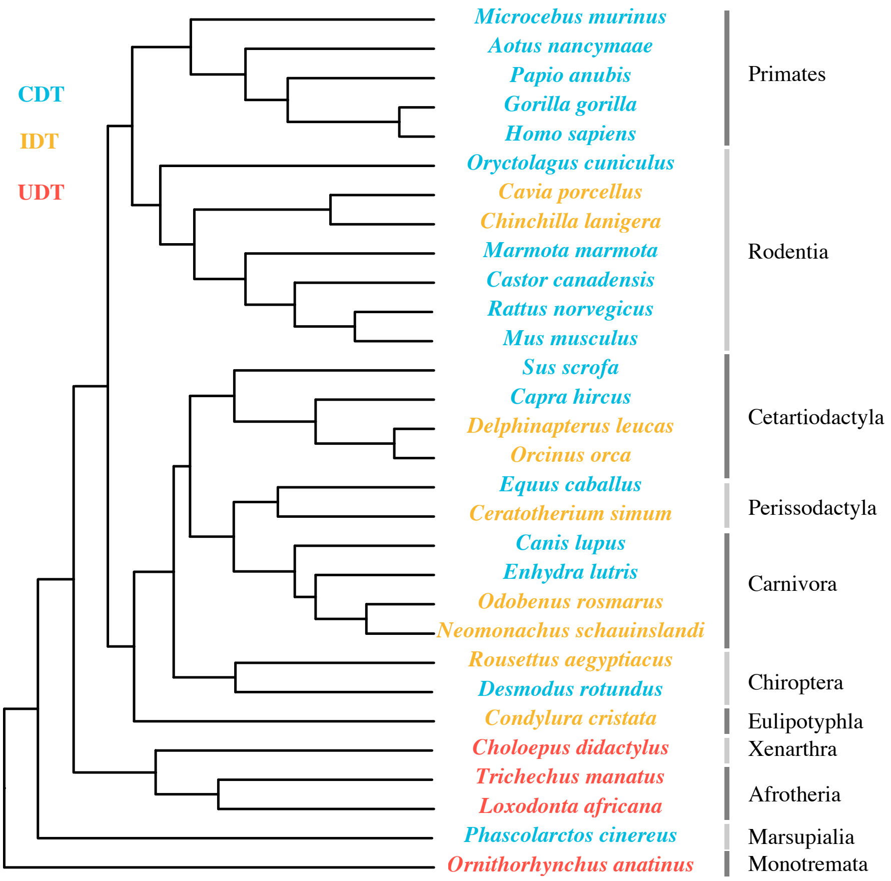

Figure S1. The phylogeny of 30 mammals used in CAFE (and in other subsequent evolutionary analyses such as PAML) using TimeTree (http://www.timetree.org/).

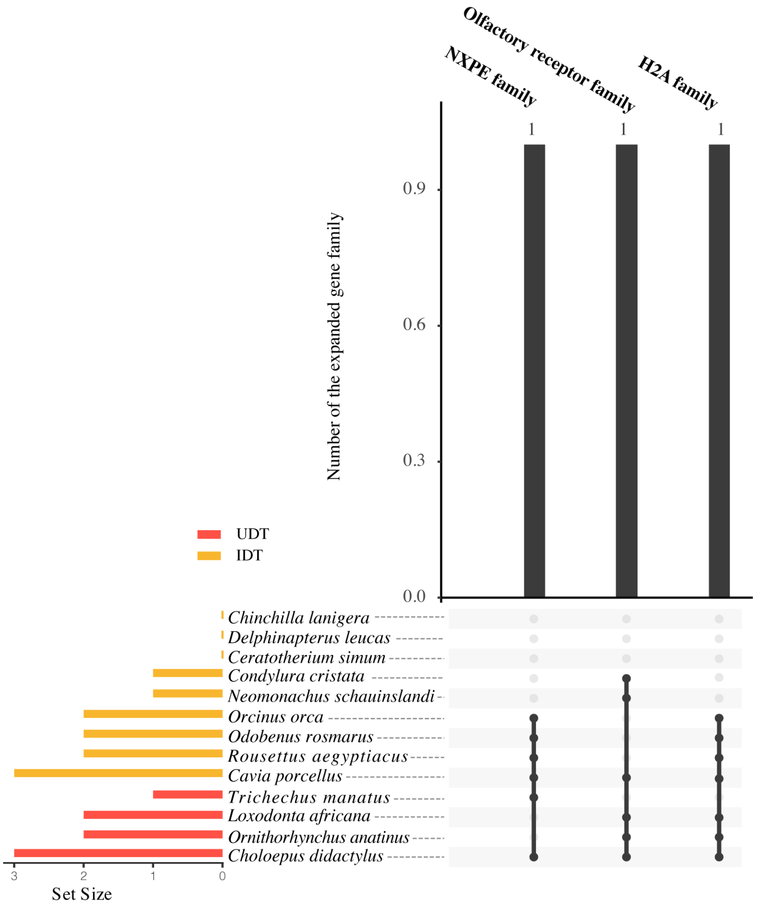

Figure S2. The expanded gene family shared in ascrotal mammals.

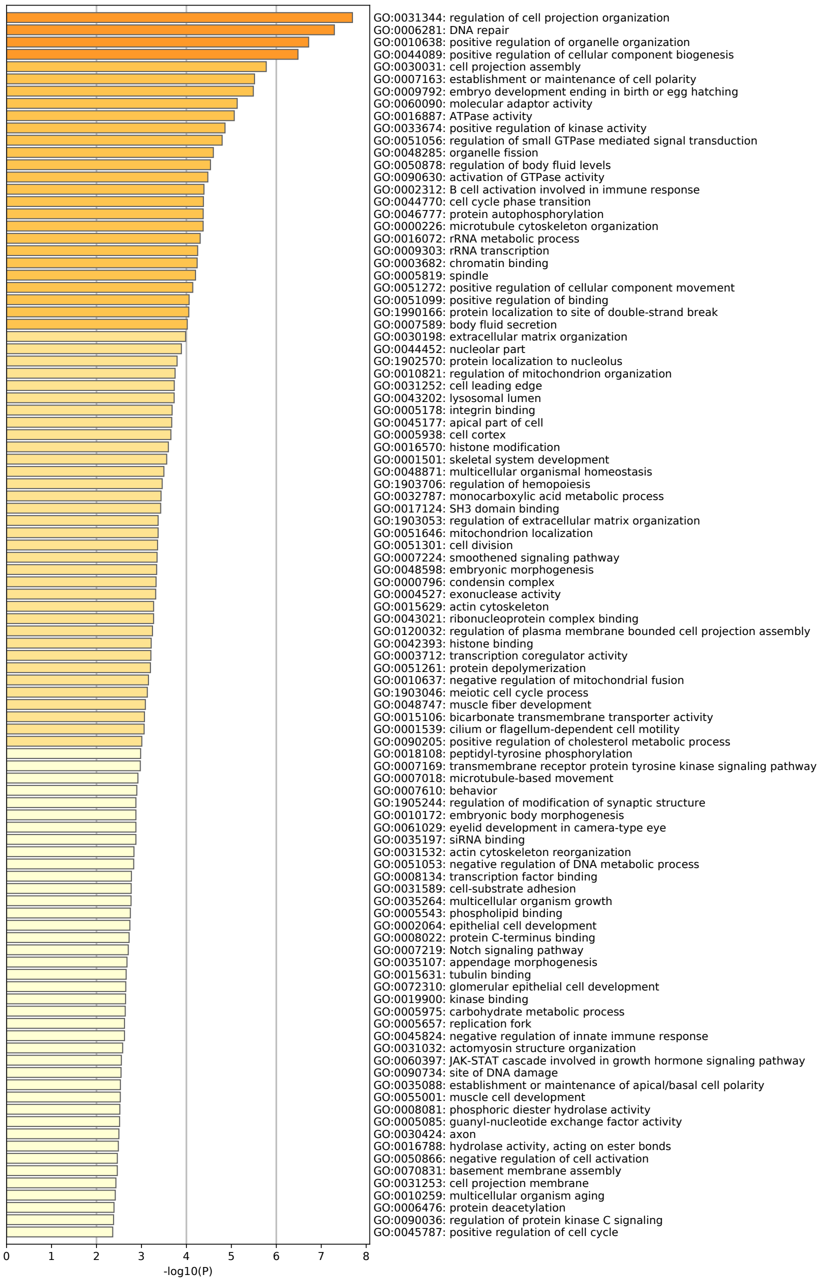

Figure S3. Heatmap of Top100 GO enrichment terms of 589 genes containing UDT mammal-specific amino acid substitutions.

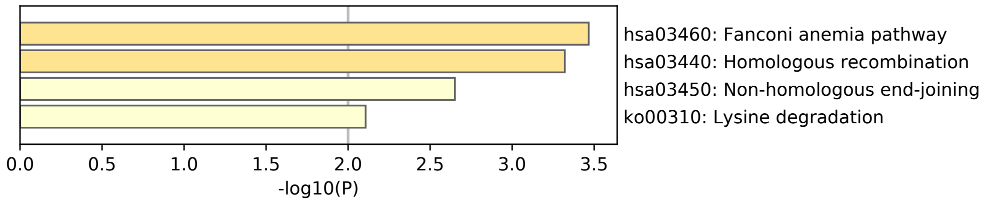

Figure S4. Heatmap of KEGG pathway enrichment terms of 589 genes containing UDT mammal-specific amino acid substitutions.

LAMB1 H979N

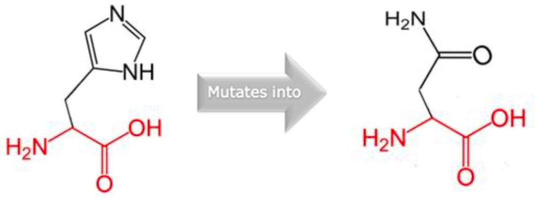

Histidine Asparagine

- The mutant residue is smaller, this might lead to loss of interactions.
- The mutation introduces an amino acid with different properties, which can disturb this domain and abolish its function.
- The wild-type residue is annotated in UniProt to be involved in a cysteine bridge, which is important for stability of the protein. Only cysteines can make these type of bonds, the mutation causes loss of this interaction and will have a severe effect on the 3D-structure of the protein.
- Together with loss of the cysteine bond, the differences between the old and new residue can cause destabilization of the structure.

Figure S5. Functional prediction of LAMB1 H979N.

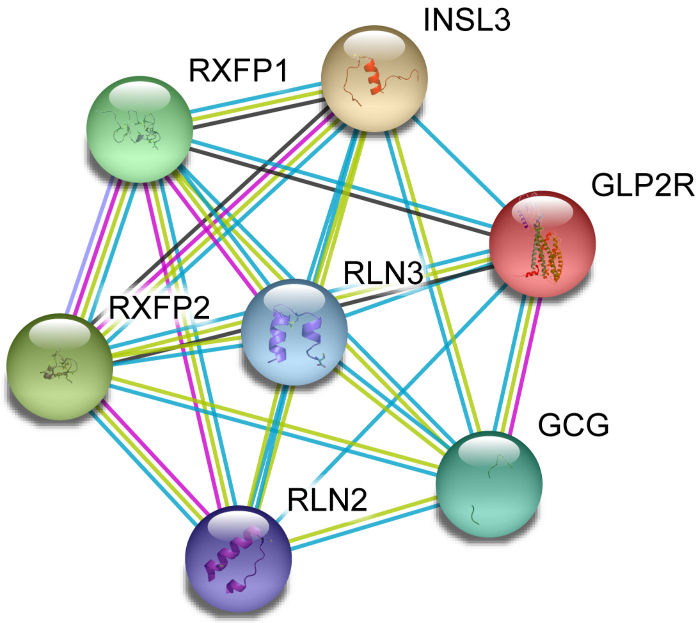

Figure S6. Interaction between GLP2R and INSL3 from String (https://version11.string-db.org/).

GLP2R M256V

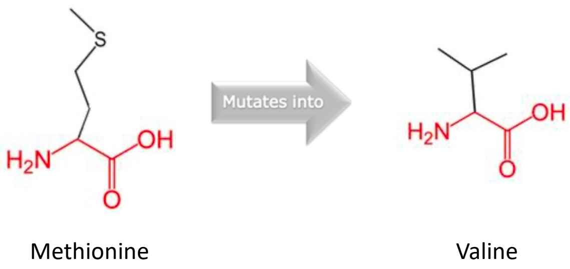

- The mutant residue is smaller, this might lead to loss of interactions.
- The mutated residue is located in a domain that is important for the main activity of the protein. Mutation of the residue might disturb this function.

Figure S7. Functional prediction of GLP2R M256V.

SPATA5 H811R

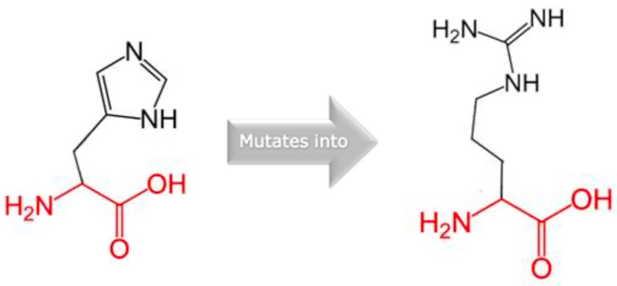

Histidine Arginine

- The mutant residue is bigger than the wild-type residue.
  The wild-type residue charge was NEUTRAL, the mutant residue charge is POSITIVE.
- This mutation was observed more often at this position in other homologous sequences. This means that more proteins exist with that mutant residue than with the wild-type residue. It is probably unlikely that your mutation of interest will be damaging for the protein.

Figure S8. Functional prediction of SPATA5 H811R.

TESK1 N332I/S

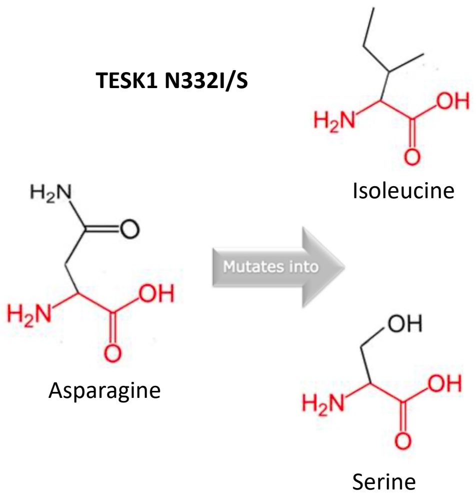

Isoleucine:

- The mutant residue is smaller than the wild-type residue.
- The mutant residue is more hydrophobic than the wild-type residue.
- Your mutant residue was not among the residue types observed at this position in other, homologous sequences which might indicate that the mutation is possibly damaging to the protein.
- The mutation introduces a more hydrophobic residue at this position. This can result in loss of hydrogen bonds and/or disturb correct folding.

Serine:

- The mutant residue is smaller than the wild-type residue.
  The mutant residue is more hydrophobic than the wild-type residue.
- The mutated residue is located in a domain that is important for the main activity of the protein. Mutation of the residue might disturb this function.
- The mutation introduces a more hydrophobic residue at this position. This can result in loss of hydrogen bonds and/or disturb correct folding.

Figure S9. Functional prediction of TESK1 N332S/I.

MLXIPL Q716R

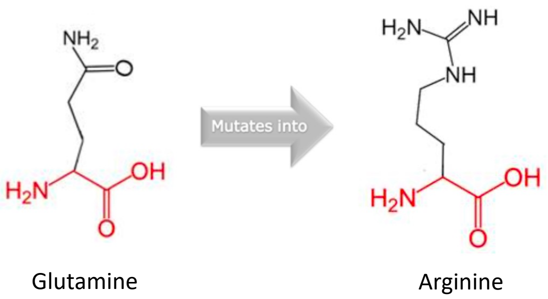

- The mutant residue is bigger than the wild-type residue, this might lead to bumps. The wild-type residue charge was NEUTRAL, the mutant residue charge is POSITIVE.
- The mutation is located within a stretch of residues annotated in UniProt as a special region: Leucine-zipper. The differences in amino acid properties can disturb this region and disturb its function.
- The mutation is located near a highly conserved position.

Figure S10. Functional prediction of MLXIPL Q716R.

Hope Version 1.1.1

ABL1 P997Q

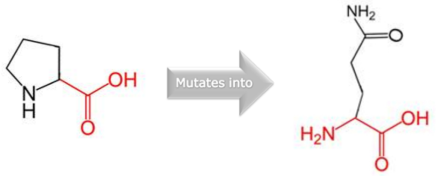

- The mutant residue is bigger and less hydrophobic than the wild-type residue.
- The mutation is located within a stretch of residues annotated in UniProt as a special region: F-actin-binding. The differences in amino acid properties can disturb this region and disturb its function.
- The wild-type residue is a proline. Prolines are known to be very rigid and therefore induce a special backbone conformation which might be required at this position. The mutation can disturb this special conformation.

Figure S11. Functional prediction of ABL1 P997Q.
